# Source localization using virtual magnetoencephalography helmets: A simulation study toward a prior-based tailored scheme

**DOI:** 10.3389/fnins.2022.947228

**Published:** 2022-09-06

**Authors:** Oshrit Arviv, Yuval Harpaz, Evgeny Tsizin, Tal Benoliel, Dana Ekstein, Mordekhay Medvedovsky

**Affiliations:** ^1^Department of Neurology, Agnes Ginges Center of Human Neurogenetics, Hadassah Medical Center, Jerusalem, Israel; ^2^Faculty of Medicine, Hebrew University of Jerusalem, Jerusalem, Israel; ^3^The Leslie and Susan Gonda Multidisciplinary Brain Research Center, Bar-Ilan University, Ramat Gan, Israel; ^4^The Zandman-Slaner Graduate School of Engineering, Tel Aviv University, Tel Aviv, Israel

**Keywords:** source estimation, source localization, inverse problem, equivalent current dipole, dipole fit, gain matrix, epilepsy surgery

## Abstract

Magnetoencephalography (MEG) source estimation of brain electromagnetic fields is an ill-posed problem. A virtual MEG helmet (VMH), can be constructed by recording in different head positions and then transforming the multiple head-MEG coordinates into one head frame (i.e., as though the MEG helmet was moving while the head remained static). The constructed VMH has sensors placed in various distances and angles, thus improving the spatial sampling of neuromagnetic fields. VMH has been previously shown to increase total information in comparison to a standard MEG helmet. The aim of this study was to examine whether VMH can improve source estimation accuracy. To this end, controlled simulations were carried out, in which the source characteristics are predefined. A series of VMHs were constructed by applying two or three translations and rotations to a standard 248 channel MEG array. In each simulation, the magnetic field generated by 1 to 5 dipoles was forward projected, alongside noise components. The results of this study showed that at low noise levels (e.g., averaged data of similar signals), VMHs can significantly improve the accuracy of source estimations, compared to the standard MEG array. Moreover, when utilizing a priori information, tailoring the constructed VMHs to specific sets of postulated neuronal sources can further improve the accuracy. This is shown to be a robust and stable method, even for proximate locations. Overall, VMH may add significant precision to MEG source estimation, for research and clinical benefits, such as in challenging epilepsy cases, aiding in surgical design.

## Introduction

It is well-known, since the first publication of human scalp EEG by Berger (1929), that synchronized neuronal activity can produce electric field changes, recordable from the human scalp. Thirty-nine years later, when David Cohen published the first magnetoencephalography (MEG) recording (Cohen, 1968), it became clear that the weak magnetic fields produced by a group of neurons can be measured outside the human head. However, the constraint-free inverse problem of estimating sources of EEG or MEG signals is ill-posed, and accordingly there are still ongoing debates and emerging new approaches (Baillet, 2010; Supek and Aine, 2019).

Spatial sampling of the neuroelectromagnetic fields is a key element in source estimation (Supek and Aine, 2019). This topic has recently regained interest, with the development of new MEG technologies, particularly optically pumped magnetometers (Iivanainen et al., 2019, 2021; Tierney et al., 2020). Ideally, subscribing with the Nyquist-Shannon sampling theorem, the spatial Nyquist frequency of the MEG sensor array should be above the highest spatial frequency of the brain signal. Thus, increasing sensor density can improve the sampling. However, increasing the density above a certain limit (defined by the spatial Nyquist frequency) does not add more information, and will only result in a higher device cost. This was shown for both EEG (Srinivasan et al., 1998; Song et al., 2015) and MEG (Vrba et al., 2004). Nonetheless, a MEG device can be supplemented with sensors at different angles and distances from the scalp, improving the spatial sampling of magnetic fields (Nurminen et al., 2010, 2013), while alongside increasing the device complexity and cost.

**FIGURE 1:**
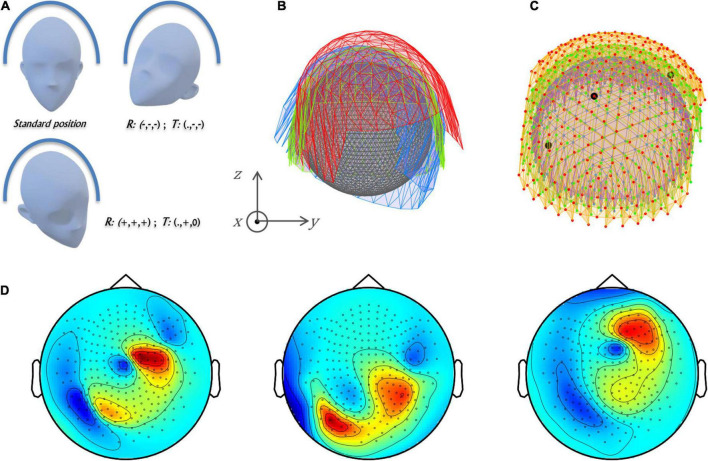
Virtual MEG helmet construction and source model explained. **(A)** The translations [T] and rotations [R] of the head of the subject inside the MEG helmet translate to **(B)** the counter movements of the MEG sensor arrays while the head remained static. In part I simulations, the coordinates of the sensors were translated (15, 15, 15) mm and rotated in (20 20 20)° (red) as well as (–15, –15, 0) mm and rotated in (–20, –20, –20)° (blue) in relation to the standard helmet position (0, 0, 0) (green). The combination of green and red MEG arrays represents VMHa, while the combination of all three arrays represents VMHb. The spherical head model positioned inside the VMH is portrayed in gray. **(C)** A three-layer spherical grid (10 mm between layers), covering the volume of a spherical head model (inner layer: purple, outer layer: orange, middle: lime). Three black dots mark the locations of three dipoles within the grid in a particular simulation out of the 1,000 performed that consisted of three dipoles. **(D)** Sensor space representations as 2D topographical maps of the forward projections of all three dipoles presented in panel **(C)**. Each map presents a 248 MEG array: left map–standard helmet [green in panel **(B)**], middle map—red MEG array in panel **(B)**, right map—blue MEG array in panel **(B)**.

MEG measures weak magnetic fields, mainly generated by post-synaptic dendritic currents in cortical pyramidal cells. The sensor signals contain a mixture of contributions from multiple brain sources (Baillet, 2010; Supek and Aine, 2019). The spatial frequencies of the measured magnetic fields depend on the distances between sources and sensors. Deeper sources are associated with magnetic fields characterized by lower spatial frequencies. When several sources are simultaneously active in the brain, different components of the complex field may have different spatial frequencies, yet they will be picked up with minimum distortion by changes in conductivity of the different head tissues: brain, scull, and scalp. This is a benefit over EEG, which is strongly influenced by these structures, manifesting distortions (Sarvas, 1987; Hansen et al., 2010). However, one of the shortcomings of MEG is that the MEG sensors are not fixed to the scalp, in contrast to EEG, and head movements can lead to source estimation inaccuracy. Yet, this ostensible disadvantage can in fact be used for improvement of the spatial sampling of the neuromagnetic fields—if the head position is measured, and utilized for generating a virtual MEG helmet (VMH) (Medvedovsky et al., 2016). That is, a virtual helmet can be constructed, if the same type of activity is recorded using MEG in different head positions and then, transforming the multiple head-MEG coordinates into one head frame. Thus, one MEG position and multiple head positions, is converted to one head position with multiple MEG positions. Treating the brainwaves measured at the different MEG positions (relative to the head) as if they were recorded simultaneously, resembles using a MEG device with twice, thrice or N times more channels, depending on the N head positions (Figure 1). The constructed VMH has sensors placed in different distances and at different angles relative to the sources of brain activity, implying that the spatial sampling of the neuromagnetic fields might be richer in comparison to a standard MEG recording. Indeed, it has been shown that VMH can increase the total information, as opposed to a standard MEG helmet (Medvedovsky et al., 2016). However, whereas total information is an important figure of merit (Shannon, 1949), until now the source localization accuracy of VMH and of standard MEG helmet have not been compared. Notably, finding the optimal settings for constructing a VMH is a non-trivial task, as alongside the particular enrichment of the spatial sampling by the fuller sensor layout of VMHs, in comparison to the standard MEG helmet, the measured noise relative to the signal of interest increases (Medvedovsky et al., 2016).

The research questions addressed by this study are whether the VMH scheme can improve source estimation accuracy as opposed to a standard MEG helmet, without (simulations part I and II) and with (simulations part III) the use of localization information, gained by previous MEG recordings. To this end, series of controlled simulations were carried out. Several VHMs were constructed by applying various combinations of rotations and translations. Since in simulations, the source model characteristics (e.g., locations, orientations, and noise levels) are predefined, this approach was chosen as a direct way to evaluate and compare the source estimation accuracy between a standard MEG helmet and the constructed VMHs. In the simulations of part I, the VMH method was examined for quality of source estimations, given different number of neural generators and levels of noise. In the simulations of part II, the robustness of the VMH method was assessed, serving as theoretical grounds to the simulations carried out in part III. In part III, a tailored approach to VMH was investigated. Assuming a previous MEG recording, it was examined whether tailoring the constructed VMH to the postulated set of sources is an advantageous approach.

## Materials and methods

Simulations and analyses were performed using MATLAB 2020a (The MathWorks) and FieldTrip open-source toolbox for Advanced MEG Analysis (Oostenveld et al., 2011).

### Construction of virtual helmets

A model based on a whole-head, 248-channel magnetometer array (4-D Neuroimaging, Magnes 3,600 WH) was used in the controlled simulations. The head shape and position were taken from a previous technical work: calibration of the MEG device at the Electromagnetic Brain Imaging Unit at Bar-Ilan University; no experiment was conducted. A standard head position inside the MEG helmet was defined according to a healthy adult male (one of the authors of this article) in a fully supine posture. [Comment: in the standard position the coordinates system of the physical helmet is such that they approximately align with the head’s planes of view: axial–(x, y) plane, coronal–(y, z) plane, sagittal–(x, z) plane].

In order to construct VMHs, the guided movements of the subject head situated inside a MEG helmet were emulated by the counter movements of the standard physical MEG array. Each recording section in a specific head position relative to the physical MEG array was simulated by the corresponding counter translations and rotations of the MEG array. Consecutively, while a standard MEG helmet consisted of a single 248-channel array, the VMHs consisted of a combination of *n* MEG arrays, and hence a 248*n channels, *n* > 1 (n is the number of head positions that were virtually simulated by the translated and rotated MEG array). In the current study, the VMHs were constructed by combing two or three 248-channel MEG arrays (*n* = 2 or 3), one located in a standard position, and the other(s) undergoing a combination of translations and rotations (Figure 1B). All rotations and translations were performed relative to the (0, 0, 0) point in head coordinates [i.e., the head was fitted with a single sphere, and the origin of this sphere was set to the (0, 0, 0) position in the head coordinate system]. The rotations were carried out prior to translations, in order to rotate around an axis passing through the origin (translations: ±15 mm or 0 mm in each of the x/y/z directions and, rotations: ± 20° or 0°around each of the x/y/z axes). The magnitude of the translations and rotations were chosen according to the limitations of physical anatomy and the fitted spherical head model (*r* = 95.5 mm) as well as the spatial constraints of the standard MEG helmet (e.g., an upward head translation, in the –Z direction, is not feasible in all VMHs). Consequently, if a VMH was found to clash with the head model, it was excluded from simulations—four constructed VMHs were excluded. A full list of the concluding VMHs appears in Table 1.

**TABLE 1:** A list of the examined VMHs in part II and III.

Serial no. of the VMH or standard	Number of MEG arrays	Rotations (°)	The effective rank of gain matrix
		Translations (mm)	
1–Standard	1	–	214
		–	
2	2	(20, 0, 0)°	296
		(–15, 15, 0) mm	
3	2	(0, 20, 0)°	305
		(–15, 15, 0) mm	
4	2	(0, 20, 0)°	306
		(–15, –15, 0) mm	
5	2	(0, 0, 20)°	302
		(–15, –15, 0) mm	
6	2	(–20, 0, 0)°	300
		(–15, –15, 0) mm	
7	2	(0, –20, 0)°	285
		(–15, 15, 0) mm	
8	2	(0, –20, 0)°	286
		(–15, –15, 0) mm	
9	2	(0, 0, –20)°	297
		(–15, 15, 0) mm	
10	2	(0, 0, –20)°	299
		(–15, –15, 0) mm	
11–VMHa	2	(20, 20, 20)°	293
		(15, 15, 15) mm	
12	2	(20, 20, 20)°	303
		(–15, –15, 0) mm	
13	2	(–20, –20, –20)°	295
		(–15, –15, 0) mm	
14–VMHb	3	(20, 20, 20)° (–20, –20, –20)°	352
		(15, 15, 15) mm (–15, –15, 0) mm	

Each VMH was assigned with a serial number. The list differentiates by the number of combined MEG arrays, the rotations and translations involved in the construction of these VMHs and the effective rank of their gain matrices, as determined using SVD.

### Source model

Using the head shape, a three-layered spherical grid was defined, with 10 mm between layers, and 8–12 mm in-between points comprising each layer (642 equally spaced points per layer, accordingly, the distances between grid points in the outer spheres are larger than in the inner sphere). Points outside the head or below the ears [(0, ±10, 0) mm from edges] were ignored, leaving 1,084 grid points covering the spherical head model (Figure 1C).

For each grid point, a dipole was defined by two orthogonal vectors that were tangential to the layer surface. Since a single sphere was used as a head model, no vectors were defined normal to the layer surface. The amplitude of each vector was randomly chosen between half to a unit vector.

### Forward model

In each series of simulations, one or several dipoles were randomly placed within the three-layer spherical grid. Once randomly selected, the same set of dipoles (locations and orientations) were applied when comparing between different MEG arrays (standard MEG helmet and various VMHs), in order to maintain fair evaluations. The forward projections of magnetic fields were computed using FieldTrip (Oostenveld et al., 2011), while applying a homogenous spherical conductor model (Cuffin and Cohen, 1977). Gain matrices of 248 (number of MEG channels) for a standard sensor array, or 248*n (when *n* is number of multiplies of MEG arrays included in the VMHs) for VMHs by 2,168 (number of vectors in the source space—1,084 grid points multiplied by two orthogonal orientations), were formed.

The rank of the gain matrix, the number of degrees of freedom, is of interest, as it can characterize the general impact of a particular VMH on source localization, without the impact of specific neural activation patterns, noise, and the parameters of the inverse model. The effective rank of gain matrices (for *n* = 2, Table 1) was determined using SVD (singular-value decomposition). The rank of the gain matrix is equal to the number of non-zero singular value of the SVD of the gain matrix. However, the effective ranks are calculated from the truncated gain matrices, by assuming singular values that are greater than a tolerance value are equivalent to zero, hence, capturing the number of independent channels (leadfields) that are of significant effective influence (Lindfield and Penny, 2019). The tolerance value was set to 0.1% of the maximal singular value (Nurminen et al., 2010).

### Noise model

Alongside the implanted sources, two types of noise components were added to the MEG channels, in a 1 to 2 ratio: technical noise (TN) and brain noise (BN). It is assumed that magnetic noise originating from the surroundings was thoroughly cleaned and there are no non-physiological noise sources within the subjects (e.g., metallic dental work). The TN and BN noise components were added to the simulated fields, such that the noise levels of the overall noise (ON) were set to particular fractions of the signal of interest: 0.1 or 0.3 (other noise levels are discussed in Supplementary material).

#### Technical noise

An uncorrelated noise across the sensors, which accordingly does not depend on the head position inside the MEG helmet or on the set of neuro-electromagnetic sources. Simulating TN of the system was obtained by assigning a Gaussian random noise to each sensor of the standard helmet or of the combined sensor arrays of VMHs.


(1) $$\begin{array}{c} T\,N∼k*N\,{\left(0,1\right)}_{o\,v\,e\,r\,s\,e\,n\,s\,o\,r\,s}\\ w\,h\,e\,r\,e:k=f*\frac{1}{3}*\bar{s\,t\,d\,\left(s\,i\,g\,n\,a\,l\right)} \end{array}$$

In order to scale the added TN to the signal of interest, the random noise is scaled by a constant factor, *k*, which consist of: (1) the noise level factor, *f*, determining the relative proportion to the signal of interest (e.g., 0.1 or 0.3); (2) a 1/3 for assigning the relative weight of the TN from ON, taking into account that std(TN) = std(ON)/3; and (3) the mean of STD of a simple model of the signal of interest. In order to assess the 3*^rd^* factor, the signal of interest was modeled by conducting 1,000 iterations of randomly distributing three simultaneously active unit dipoles throughout the grid. For each iteration, a forward solution only in the standard MEG helmet was obtained, and its STD was calculated. In contrast to the BN component (as will be explained bellow), the TN is indifferent to the particular VMH, hence, the degree of TN for the standard MEG array and for all the other MEG arrays of the VMHs should be the same, while at the same time the SNR should vary between the different sensor arrays of the VMHs per each set of sources. Thus, the obtained mean of the STDs for the standard MEG helmet (multiplied by a weight of 0.75: the mean of the signal ranges from 0.5 to 1) was used to scale the TN. Furthermore, as will be explained bellow, the relative scaling of TN to BN was set by the standard head position alone.

#### Brain noise

A correlated noise component across MEG channels, which is a result of the activity of multiple neuronal circuits in the brain, different than the signal of interest. BN depends on the head position inside the helmet and therefore cannot be of the same magnitude on the different sensor arrays of the VMH. Simulating BN was obtained by assigning a Gaussian random noise to the sources (i.e., to each of the three-layer grid positions within the brain volume), then scaling this random noise by a constant factor to its proportion of ON, and last, using forward calculation (multiplying by the gain matrix of the VMH) to create correlated brain background noise on the sensors.


(2) $$\begin{array}{c} B\,N∼G\,a\,i\,n\cdot \left(c*N\,{\left(0,1\right)}_{o\,v\,e\,r\,g\,r\,i\,d}\right)\\ w\,h\,e\,r\,e:c=\frac{2*s\,t\,d\,\left(T\,N\right)}{\bar{s\,t\,d\,\left(B\,N\,s_{s\,t\,a\,n\,d\,a\,r\,t\,h\,e\,l\,m\,e\,t}\right)}} \end{array}$$

The BN is sensitive to the dispersal of the noise at the sources relative to the sensor arrays, and thus depend on the particular VMH. In order to scale between the relative contributions of TN and BN to the ON, the standard head position was used. Accordingly, the random noise at the sources of BN was multiplied by two times the std(TN) and divided by the mean of STD of calculated BNs at the standard helmet, all constant factors. The latter, in practice, was obtained by running 1,000 iterations, in which random vectors (drawn from a Gaussian distribution) were associated with all simulated brain sources (grid points), and using forward model (multiplying on the gain matrix), the BNs on the sensors of the standard MEG helmet were calculated. Hence, a distribution of BNs was obtained, and the mean STD of simulated BNs was calculated. Using these constant factors, obtained from the standard helmet, to scale the particular sources of BN per each simulation—the assigned random noise over the grid, directed the std(BN) = 2*std(TN) relation. Next, the VMH construct is taken into account, as the scaled sources of BN are forward projected over the particular VMH’s sensors.

Additionally, as potential MEG recording time in each head position decreases in proportion to the number of positions, there is an expected decrease in number of recorded events that are averaged per head position (that is per each MEG array of VMHs) (Medvedovsky et al., 2016). In order to take this into account and assuming the accumulated events are evenly distributed between recordings in different head positions, the ON was multiplied by the square root of n, the number of MEG arrays in VMHs.


(3) $$O\,N=\sqrt{n}*\left(T\,N+B\,N\right)$$

### Inverse model

A single equivalent current dipole (ECD) model (Hämäläinen et al., 1993) is a simple and widely accepted way of inverse modeling in MEG. However, for estimation of complex sources, single ECDs can be applied in sequential steps. In the current study, each simulated field was source estimated by sequential single ECD fit, applied as following:

At first the single ECD was fitted to the simulated field using the symbolic matrix left division operator in MATLAB, \, dividing the elements of the gain matrix corresponding to each location in the three-layer spherical grid (i.e., with one location two orthogonally oriented unit vectors were associated) by the measured field (the left division command in MATLAB uses QR decomposition with pivoting to find the pseudo-inverse).


(4) $$L_{i}={\left(G\,a\,i\,n_{i}\right)}_{l\,e\,f\,t}^{-1}\,M_{s\,i\,m}$$

where *L*_*i*_ is the solution of the ith location represented by a two by one vector of the dipole source estimate; *Gain*_*i*_ is a 248*n by two matrix corresponding to the dipole of the ith iteration; and *M_*sim*_* is a 248*n by one vector corresponding to the simulated neuromagnetic field.


(5) $$M_{e\,s\,t_{i}}=G\,a\,i\,n_{i}\,L_{i}$$

(6) $$R_{i}=c\,o\,r\,r\,\left(M_{s\,i\,m},M_{e\,s\,t_{i}}\right)$$

The location related with the highest squared spatial correlation between the original and estimated fields was chosen. Then, the field linked to this ECD (*M*_*est_i_max*_) was subtracted from the original field. Successively, the next single ECD was fitted to the residual field. This procedure was repeated six times. Dipoles with a magnitude (Euclidean norm) that is proportionally weaker than a threshold of 0.3 of the strongest dipole were discarded (for details regarding the choice of 0.3 threshold, please see Supplementary Figure 1–top row and Supplementary material).

Sequential single ECD fit has been used with some methodological variations both in basic research (Salmelin, 2010) and in epileptiform activity source estimation for epilepsy surgery (Medvedovsky et al., 2012). In the present article, we refer to this method as sequential dipole fit (SDF). It is important to note that often the single ECD is fitted after the sensors were selected over the field maxima. Here, no sensors were selected a priori, in order to simplify the handling of complex randomly distributed sources, and since the way of sensors selection in VMH is not yet defined and requires further study. While not-selecting sensors can influence the dipole fit accuracy, the dipole fits in VMHs and standard MEG helmet were carried out in the same way, thus not affecting the comparison between them.

### Quantitative assessment of source localization

At each simulation, which compares between the different VMHs and a standard MEG helmet, the placed set of dipoles: their number, locations, and directions (expressed by amplitude relation of the two associated orthogonally oriented vectors) were identical. To compare the source estimations, for each simulation, the mean distance accuracy between the locations of the placed and solved dipoles was calculated. Placed dipoles without a matching solution, and solved dipoles that are superfluous, did not contribute to the calculated distance accuracy. The number of solved dipoles, in comparison to the number of placed dipoles, was also used as an indicator of the success of source estimations.

Given a series of different VMHs and a particular set of placed dipoles, a standard helmet or a specific VMH were declared as successful in their source estimations, offering the best solution, according to the following scheme: (1) is the number of solved dipoles identical to the number of placed dipoles? (2a) if so, find the helmet that gave the lowest distance error. (2b.i) if not, is the number of solved dipoles smaller by one missing dipole? If so, repeat 2a. (2b.ii) if not, is the number of solved dipoles larger by one superfluous dipole? If so, repeat 2a. Etc. This scheme gave precedence to the number of located dipoles over distance accuracy, as well as to missing dipoles over superfluous ones (particularly important for clinical uses).

The robustness of successful source estimations by the standard MEG helmet or a VMH was tested by inserting small fluctuations to the locations and orientations of simulated sources, and reassessing source estimations. This was performed to avoid chance successes for a particular position and orientation of simulated dipoles. It was accomplished by the following procedure: dipoles were placed at neighboring locations close to the investigated dipoles, with slightly jittered orientations (change in magnitudes were randomly selected in the range of –20 to +20% relative to originally placed dipoles). The neighbors of a grid point included the nearest grid points in its layer, and its neighbors’ nearest neighbors, as well as an additional neighbor from each of the other layers, accumulating between 4 and 20 close neighbors (14.7 ± 4.7 mm) per grid point. Like the originally placed dipoles, the characteristics of the neighboring dipoles, that is their amount, locations, and orientations, were identical at each of the compared simulations. The source estimation accuracy of these new placed dipoles was examined by the same series of VMHs and by the standard helmet as the original dipoles, and the degree of consistency in obtaining a successful solution by each specific helmet served as a measure of the robustness of that helmet to localize dipoles within a given area. This ability of a helmet was calculated by the following metric: the percentage of neighbors in which the specific helmet (VMH or standard) offered the best solution out of all examined helmets. Subsequently, this metric was also utilized in personalizing or pre-selecting a specific helmet, according to prior estimations of source locations.

### Statistical analysis

Two-way ANOVA was used to examine the effect of both the number of combined MEG arrays and the number of placed dipoles on the number of solved dipoles or the distance error. It was also used to examine the effect of threshold, or noise level. One-way ANOVA was used to examine the effect of a particular VMH or the standard helmet (as listed in Table 1) on the number of solved dipoles or the distance error. This same analysis was performed using two-way ANOVA after adding a categorical variable dividing the cases in which a helmet (Table 1) offered the best solution for the originally placed dipole pairs and the cases in which the helmet was not the best. One-way ANOVA was also used in the 3rd part of simulations to examine the effect of the helmet [the standard MEG helmet, the single best VMH (no. 3, Table 1) and the prior-based personalized VMH] on the number of solved dipoles or the distance error. Specific comparison between the standard MEG helmet and the prior-based personalized VMH was performed using t-test. When ANOVA tests were carried out, *Post hoc* comparisons were carried out by the Tukey’s Honest-Significance test. A permutation test was performed when comparing the distributions of the distance between dipoles in placed pairs for cases that there was a favorable VMH which offered best solution vs. cases there was no such VMH. A similar permutation test was also performed when comparing the distributions of the correlations between forward projected fields over the comprising sensor arrays within VMH.

### Details of simulations

The study included three sets of simulations.

#### Simulations part I: Comparing source estimations by standard helmet and virtual magnetoencephalography helmets

In the first sequence of simulations, two type of VMHs were constructed—combining two (VMHa) and three (VMHb) 248-channel MEG arrays [VMHa: two MEG arrays, one located in a standard position and a second translated by (15, 15, 15) mm and rotated in (20 20 20)°; VMHb: three MEG arrays, the two as in VMHa and a third one, which was translated by (–15, –15, 0) mm and rotated in (–20 –20 –20)° (Figure 1B)]. In each simulation, a set of between one and five dipoles were randomly placed within the three-layer spherical grid. The magnetic fields generated by the placed dipoles were forward projected, alongside noise components which were added (please see Section “Methods: Noise model”). Per each specific number of placed dipoles (1 to 5) and noise level (no noise, 0.1 or 0.3) at each sensor array (standard, VMHa or VMHb), 1,000 simulations were carried out. Source estimation was performed on the projected magnetic fields using sequential single ECD fit as described in Section “Methods: Inverse model” and the results from both VMHs and standard helmet were compared.

#### Simulations part II: Examining the robustness of successful source estimations by virtual MEG helmets

In the second sequence of simulations, a set of various VMHs were constructed (the constructed VMHs are listed in Table 1). In this part, the scenario of two active sources was further investigated: 100 pairs of randomly placed dipoles, at 0.1 noise level, were subject to source localizations by all VMHs. The magnetic fields of these dipoles were constructed by forward projections and then the sources of these fields were estimated. The estimated sources were compared to original ones; based on this comparison the best MEG helmet(s) (standard or VMHs) were identified for each of the randomly placed dipole pair. Additionally, at the neighboring grid points of each dipole within the 100 dipole pairs, similar dipoles (with a jittered orientation) were placed and were used to assess the robustness of each VMH to nearby locations and orientations (for details, please see previous section on Quantitative assessment of source localization).

#### Simulations part III: Constructing a prior-based personalized virtual MEG helmet

In the third part, three consecutive series of simulations were carried out (Figure 6, displays a flow chart): [*stage 1*] simulating the 100 pairs of dipoles as before, while only the standard MEG helmet was utilized for their localization; [*stage 2*] the source estimation of stage 1 was set as a prior source distribution in the current stage. That is, the 100 prior distributions (estimated locations and orientations) obtained in stage 1 were re-localized by the whole set of VMHs (excluding the standard MEG helmet, as the source estimation was obtained by it, avoiding a bias in its favor). Additionally, similar dipoles, with slightly jittered orientations, were placed at neighboring grid points to each of the estimated sources of stage 1 and were re-localized separately. In order to determine the closest to optimal VMH for each estimated source among the examined VMHs, the sum of cases (estimated sources of stage 1 or their neighboring dipoles) of which each VMH offered the best solutions was used as an indicator to the best VMH(s) per the specific prior source distribution. If more than one VMH was associated with the larger sum of cases, and therefore offered overall equally best solutions, one of the VMHs was randomly chosen (Supplementary Figure 2); [*stage 3*] in order to test whether the chosen VMH of stage 2 improved the source estimation relative to the actual neuronal sources, the original 100 dipole pairs simulated at stage 1 were subject to source estimation by the VMH that was chosen in the 2nd stage. Next, the source estimation quality was evaluated by comparing the original pair of dipoles, to the estimated sources of stage 3.

## Results

The simulated fields were captured differently by a standard MEG array and VMH. Placed dipoles on the grid result in forward projections that may substantially vary between the MEG arrays that comprise the VMH (for example, Figures 1C,D). As portrayed in Figure 1B, the VMH has sensors placed in different distances and at different angles relative to the electromagnetic field. The spatial sampling of the fields produced by the placed dipoles by the constructed VMH is potentially richer (Figure 1D).

### Simulations part I: Comparing source estimations by standard helmet to virtual MEG helmet

**FIGURE 2:**
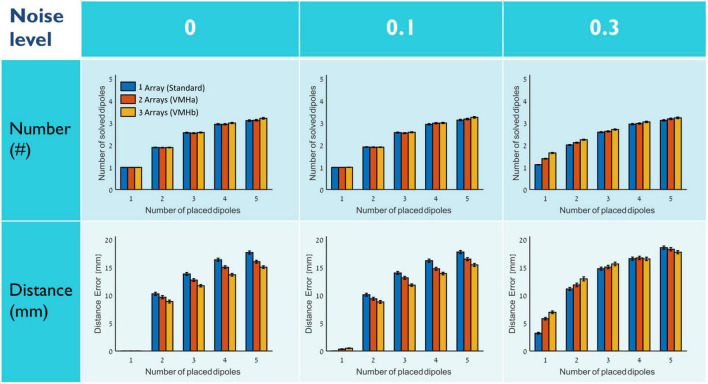
Source localization accuracy by VMHs. The number (top row) and the distance accuracy (bottom row) of solved dipoles vs. placed dipoles (between one and five dipoles) at noise levels of 0, 0.1, and 0.3. The number of combined MEG arrays in VMH is marked by color: 1 (standard)-blue, 2 (VMHa)-red, and 3 (VMHb)-yellow. Each bar represents a mean ± SEM across 1,000 simulations.

Two types of VMHs were constructed—combining two (VMHa) and three (VMHb) 248-channel MEG arrays. In each simulation, a set of between one and five dipoles were randomly placed within the three-layer spherical grid and noise components were added (for details, please see Section “Methods”). Figure 2, presents the results of the source estimation accuracy in these simulations. For fields constructed from one placed dipole, VMH did not improve source localization accuracy. For fields constructed from more than one placed dipole, the effect of the number of arrays in VMH (*n* = 1 in standard MEG helmet, and *n* = 2 or 3 in VMHa and VMHb, respectively) on the accuracy of source localization was opposite at low noise levels (0 or 0.1) in comparison to at high noise levels (0.3 or above).

At low noise levels (0–0.1), the solved sources were localized with a better distance accuracy, as the number of arrays in VMH increased. A significant interaction effect was observed between the “number of arrays” and “number of placed dipoles” [*p* < 10^–6^ and *p* < 10^–8^, for no noise and 0.1 noise levels, respectively; particularly for the same number of placed dipoles: there were significant effects for two placed dipoles, between one and three arrays *p* = 0.0030 and 0.0083 for no noise and 0.1 noise level, respectively; for three placed dipoles, between one and three arrays *p* < 10^–6^ for both no noise and 0.1 noise levels; and for four and five placed dipoles, between all number of arrays *p* was in the range of between 0.01 till 10^–6^, excluding a non-significant effect between two and three arrays (for five placed dipoles *p* = 0.18 and 0.12, and for four placed dipoles at 0.1 noise level *p* = 0.39)]. In contrast, the number of solved dipoles did not show a significant interaction effect between number of arrays and number of placed dipoles at low noise levels (no noise or 0.1). Notably, it is important to mention that even at the no noise condition (0 noise level), there are still some placed dipole combinations for which the VHMs resulted in an increase in distance error. At higher noise levels (0.3 or above), the solved dipoles were localized with an increased distance error relative to the placed dipoles, while the distance accuracy even deteriorated with higher number of arrays in VMH (Figure 2 and Supplementary Figure 1 middle row: right panels). Moreover, it was accompanied by localization of superfluous dipoles (Figure 2 and Supplementary Figure 1 middle row: left panels). For more details regarding the results of the statistical tests, please see Supplementary material.

Overall, part I set of simulations demonstrated that VMHs can improve source localization accuracy for fields that cannot be explained by a single ECD when noise levels are low.

### Simulations part II: Examining the robustness of successful source estimations by virtual MEG helmets

As VMHs offer a richer spatial sampling of the magnetic fields, the variations in distances and angles of the MEG sensors may influence the quality of source estimations. Following, a set of various VMHs were constructed (the constructed VMHs are listed in Table 1), and pairs of placed dipoles at 0.1 noise level were subject to source localizations by all VMHs.

**FIGURE 3:**
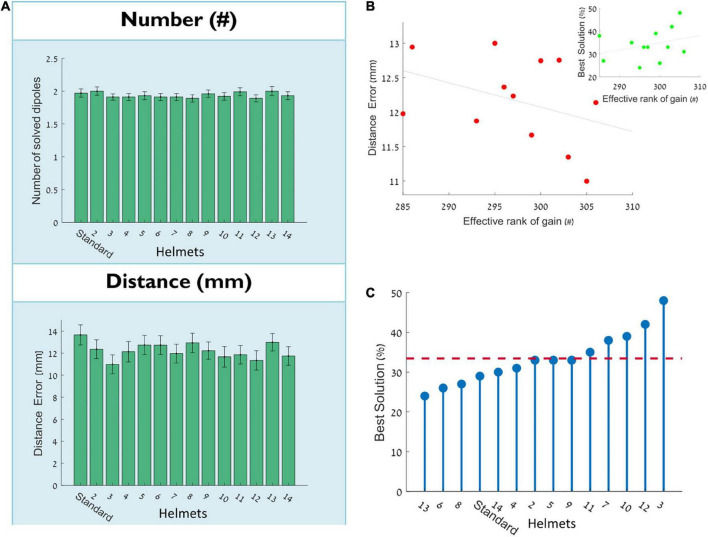
Assessing the source estimation accuracy by various VMHs. **(A)** The number of solved dipoles and the distance error: mean ± SEM of the 100 simulations of placed dipole pairs. **(B)** The relation between the mean distance error to the effective rank of the gain matrix. Each red point represents a VMH (*n* = 2, Table 1) (*R* = –0.38, *p* > 0.2) [inset: same for the percentage of best solution, as explained in panel **(C)**, green point represents a VMH (*n* = 2, Table 1) (*R* = 0.31, *p* > 0.3)]. **(C)** For each of the tested VMHs and standard helmet, displays the percentage of cases among the 100 placed dipole pairs in which the specific VMH offered the best solution of all the tested VMHs. The x-axis is sorted to emphasize the relative distribution around the mean.

In part I simulations the case of source estimations of two placed dipoles at noise level 0.1 by a two-arrays helmet was not significantly different than by a standard MEG array or three-array VMH. Consistent with this result, Figure 3A demonstrates that when all helmets listed at Table 1 are used for all dipole pairs, there is no significant difference for both the number of solved dipoles and the distance accuracy (*p* > 0.9 and *p* > 0.7, respectively), as well as no effect for the number of comprising arrays (*p* > 0.8 and *p* > 0.2, for number of solved dipoles and the distance accuracy, respectively). Figure 3B displays the relation between the effective rank of the gain matrix of each VMH (*n* = 2, Table 1 and Section “Methods: Forward model”) and the obtained distance error (*R* = –0.38, *p* > 0.2). However, the number of examined VMHs should have been larger than 50 in order to achieve at least 80% statistical power to reject the null hypothesis, given the value of the correlation coefficient, R (Kohn and Senyak, 2020). Regardless of the richness of the overall spatial sampling offered by a particular VMH, as is expressed by its truncated gain matrix’s rank, there may be an additional outlook on source estimation relating to each specific pair of dipoles, which the effective number of independent sensors of a VMH cannot reflect.

For each specific dipole pair, there could possibly be a VMH that may offer a more precise source localization, as opposed to other VMHs. Figure 3C, shows the percentage of cases among the 100 placed pairs of dipoles in which each of the tested VMHs offered the best solution (please see Section “Methods: Quantitative assessment of source localization”). Markedly, the standard helmet offered the best localization for only 29% of dipoles pairs (less than the average of all helmets 33.4 ± 6.6%), and none of the VMHs offered the best solution for more than 50% of simulated dipoles pairs (It is important to note that one dipole pair can have several VMHs that provide equally good solution). Figure 3B inset displays the relation between the effective rank of the gain matrix and the percentage of obtaining the best solution by that VMH [*R* = 0.31, *p* > 0.3, desired sample size of approximately 80 VMHs or above (Kohn and Senyak, 2020)].

**FIGURE 4:**
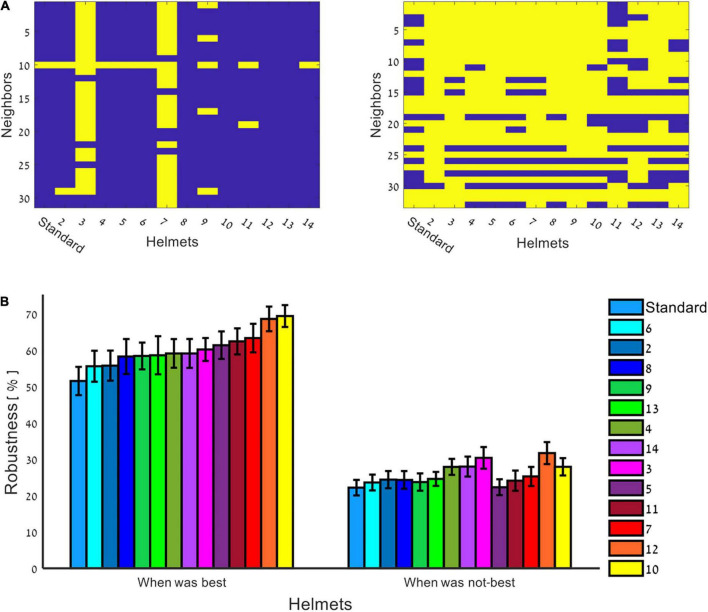
Source estimations across neighbors *via* VMHs. The neighboring grid locations of each dipole of 100 dipoles pairs were used to evaluate the source estimations by the VMHs. **(A)** Binary matrices of (VMHs, Neighbors), each of a different dipole pair: yellow–if the specific VMH offer the best (most accurate) solution, and purple–otherwise. Examples: right panel–a dipole pair which shows relatively no preference to a specific VMH, and most VMHs offered the same quality of source estimations over neighbors; left panel–a dipole pair which shows source localization preference *via* specific VMHs. **(B)** The robustness of a helmet in the source estimation of a dipole pair is represented by calculating the percentage of neighboring dipoles for which a specific helmet offered the best solution out of all helmets, while dividing into cases in which the specific helmet offered the best solution per an originally placed dipole pair (left panel), and to cases it did not for other original dipole pairs (right panel).

In order to test whether per each dipole pair the source estimation accuracy is related to matching a specific VMH to a specific dipole pair, offering potentially more appropriate spatial sampling of the generated magnetic fields, the neighboring grid locations of each dipole pair were inspected for source localization by all VMHs and standard helmet (please see Section “Methods: Quantitative assessment of source localization”). All dipoles placed at neighboring grid locations were evaluated for the helmet (one of the VMHs or standard) that offered the best solutions, and thus enabled to examine whether the successful solution of a VMH per a dipole pair is stable for nearby locations, at proximity to the originally placed dipole pairs. Figure 4A shows two examples of binary matrices of (Helmets, Neighbors), each of a different dipole pair, in which each entry is yellow if the specific helmet offers the best (most accurate) solution, and purple otherwise. The right panel demonstrates an example of a dipole pair which shows relatively no preference to a specific helmet (i.e., most VMHs offered same quality of source estimations on most of their surrounding neighbors). Alternatively, there are dipole pairs that show specific preference, and result in successful source estimation only by relying on the spatial sampling offered by specific VMHs. The left panel shows an example of such a dipole pair. Figure 4B summarizes these results, by calculating robustness: the percentage of neighbors in which each helmet offered the best solution out of all constructed VMHs and standard helmet. The bar graph is divided to cases in which the helmet offered the best solution for the originally placed dipole pair, and to cases in which the helmet was not the best. As is clearly evident, there is a significant difference between these two scenarios (*p* < 10^–10^), demonstrating that when a helmet offers the best solution for a dipole pair, it has a tendency to provide the same for the neighboring grid points and oppositely when a helmet cannot. This suggests that a good solution obtained by a particular VMH construction is a stable and robust choice per a dipole pair and across its nearby surroundings. In conclusion of this part, although there is no significant difference in the overall performance of all studied helmets, there was still a preference demonstrated by the various helmets, to more accurately estimate particular dipole pairs. The non-randomness of this preference is highlighted by the robustness of providing a good solution per a dipole pair and over its neighboring grid points by the same helmet.

### Simulations part III: Constructing a prior-based personalized virtual MEG helmet

**FIGURE 5:**
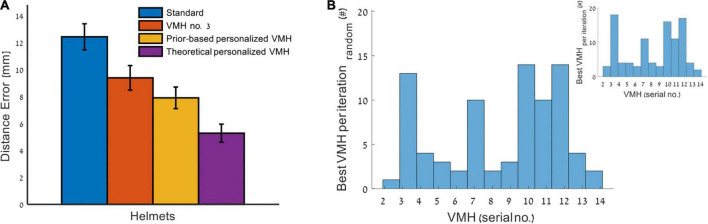
Comparing personalized VMH to standard MEG helmet. **(A)** A mean ± SEM of distance error across 100 dipoles pairs minus the excluded 18 cases where no VMH outperform the others in source estimation accuracy. The bars represent: standard helmet (blue), the overall best specific VMH (serial no. 3, see Table 1) (red), the prior-based personalized VMH (per dipole pair, the selected VMH based on simulated previous recording) (yellow) and theoretical personalized VMH (based on apriori knowledge of source locations) (purple). Statistical test was only conducted for the comparison between the standard helmet to prior-based personalized VMH, to avoid circular inference. **(B)** A distribution of the evaluated VMHs, each count represents a dipole pair for which the specific VMH was selected. For 18 dipole pairs out of 100, no VMH was selected, as the source estimation accuracy of all evaluated VMHs was the same. In cases there were more than one, yet less than all, VMH that preformed the best (Supplementary Figure 2), one VMH was randomly selected (inset: the distribution before random selection).

Figure 5A compares the distance accuracy of standard helmet (blue) to the VMH that had on average the lowest distance error and thus was the overall best single VMH for the whole 100 pairs of dipoles (VMH marked by no. 3, red, see also Table 1) to the theoretical or unfeasibly personalized VMHs, which matches to each dipole pair the VMH which gave the best solution (purple). The theoretical personalized VMH is only presented for visualization purposes, to display the possible contribution that could have been made by a VMH, if its selection was driven by an a priori knowledge of the sources’ locations. [Comment: No statistical tests were carried out, as the selection involved is biasing toward a significant effect (i.e., circular inference or “double-dipping”)]. However, one cannot completely know the identity of the neuroelectromagnetic sources. Therefore, in practice, to potentially approach the benefits of the theoretical personalized VMH (purple, Figure 5A), one can rely on a hypothesis or more specifically a prior based on previous MEG measurements, and tailor a VMH, close to the optimal, for these particular sources.

**FIGURE 6:**
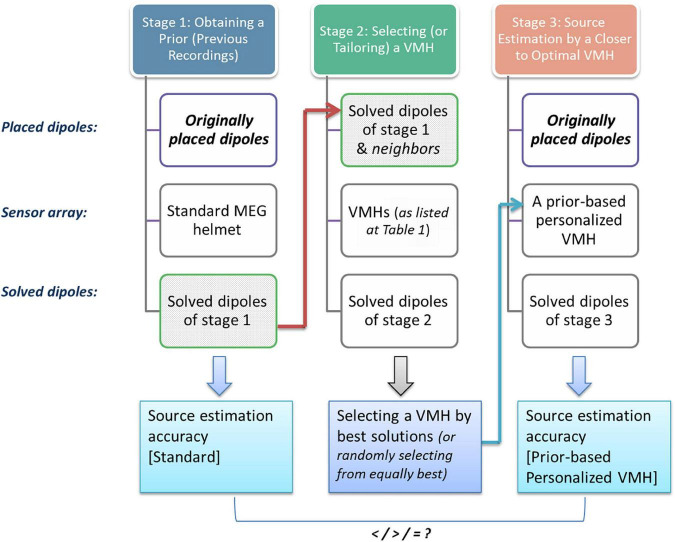
Constructing a prior-based personalized VMH. A flow chart of the sequence of simulations that were carried out in part III. (stage 1) simulating 1st set of MEG recordings in the standard position of the head inside an MEG helmet, while supposedly actual pair of active neuronal sources are imitated by a placed pair of dipoles; (stage 2) next, the source estimation of stage 1 was set as a prior source distribution in the current stage. These placed prior-based dipoles as well as similar dipoles at neighboring grid points were used to determine the closest to optimal VMH among the examined VMHs (for details, please see Section “Methods: Details of simulations: Part III”) (stage 3) following, the original active sources simulated at stage 1 are now subject to source estimation by the VMH that was chosen in the 2nd stage, and the results are compared to the source estimations of the same original sources by the standard MEG helmet. This is done in order to examine whether the chosen VMH of stage 2 advances the source estimation accuracy relative to the actual neuronal sources.

Therefore, the following methodology is presented: given previous results by MEG in a standard helmet position, the estimated sources by these recordings is set as a prior source distribution for simulating and selecting the suitable construct of a VMH (i.e., in current clinical practice, what head movements to instruct the subject in order to optimally sample the sought after neuronal sources). Here, in order to test the feasibility and effectiveness of this approach, three consecutive series of simulations were carried out (please see flow chart in Figure 6 and Section “Methods: Details of simulations: Part III”). Notably, while in practice, the simulations in stages 1 and 3 merely imitate real MEG recording, stage 2 is in its nature a simulation stage that will consist of a series of simulations also while implemented in real clinical or experimental applications, aimed to select (or tailor) a VMH that can approximate optimal results in stage 3. The chosen VMH of stage 2 is termed the prior-based personalized VMH.

Figure 5A also presents the comparison between the average distance accuracy obtained by a standard MEG helmet (blue) vs. the prior-based personalized VMH (yellow). Selecting a VMH based on a prior distribution that was obtained from previous measurements (stage 1—simulating previous MEG recordings) is shown to significantly improve source localization compared to a standard MEG helmet (significant difference for distance error *p* < 10^–5^, and a non-significant difference for number of solved dipoles *p* > 0.4). In 18 of the 100 dipole pairs tested, the set of examined VMHs offered the same quality of source estimation (Supplementary Figure 2). These cases were characterized by a significantly shorter distance between the two dipoles in the pair (*p* = 0.0001) and higher correlations between the forward projected fields of the comprising MEG arrays of the prior-based selected VMH (*p* < 0.05) (Supplementary Figure 2). Excluding these cases results in even stronger effect while comparing the prior-based personalized VMH to the standard helmet (significant difference for distance error *p* < 10^–8^, Figure 5A, and a non-significant difference for number of solved dipoles *p* = 0.3). On average, the prior-based personalized VMH (yellow) outperformed the standard MEG helmet (blue) by ∼36.4% (excluding the no-preference cases, or ∼24.8% otherwise: over all 100 dipole pairs) distance accuracy. Markedly, the single VMH which preformed best on the studied dipole pairs (no. 3, red and Table 1) also outperformed the standard MEG helmet by ∼24.5% (excluding the no-preference cases *p* < 0.05, or ∼19.5% otherwise, *p* < 0.1 marginally significant) distance accuracy. Yet, the difference in distance accuracy between the prior-based personalized VMH and the single best VMH (no. 3) was non-significant (*p* = 0.47 excluding the no-preference cases; or *p* = 0.84 otherwise). Figure 5B demonstrates the distribution of the number of times each VMH was selected across the studied dipole pairs, excluding the 18 no-preference cases (inset: before random selection, in cases there were more than one, yet less than all, VMH that preformed the best). It is clear that indeed VMH marked by no. 3 is overrepresented in the distribution, but it was not selected as most suitable in all the 100 examined dipole pairs. To avoid p-hacking a single random selection from the equally preforming helmets was applied and presented (yellow), yet 100 other random selections were also examined and the significance of the obtained results remained the same (mean distance error by the prior-based personalized helmet 10.0 ± 0.2 mm over the 100 selections, and *p* = [0.002.. 0.03] compare to standard helmet and *p* = [0.5.. 0.9] compare to best single helmet (no. 3); and when excluding the 18 non-preference cases mean distance error 7.91 ± 0.08 mm and *p* = [0.0007.. 0.0015] and *p* = [0.4.. 0.5], respectively).

In the above results, the standard MEG helmet was excluded from stage 2 and the list of helmets to select the prior-based personalized VMH, hence, avoiding the potential bias due to the role of the standard helmet in stage 1. However, to reject an assumption that the standard helmet could have had of a considerable part as a candidate helmet for stage 2, the above analyses were repeated, now also including the standard helmet. In this scenario, the standard helmet was found to perform the best above all others in only 2 of the 100 dipole pairs, and alongside some of the other helmets but not all in only three of them. As before, also in this scenario, the prior-based personalized helmet outperform the standard helmet, with a lower distance error of ∼34.6% when excluding the no-preference cases, or ∼26.6% otherwise (*p* < 10^–8^), and a non-significant difference in the number of solved dipoles (*p* > 0.1). Moreover, another validity test was as following: stage 2 was performed by solely the standard helmet, solving the forward projected fields of the localized sources of stage 1. The standard MEG helmet with the solved dipoles, preformed poorer than the standard helmet with the original dipole pairs, when calculating its source estimation accuracy relative to the original dipole pairs. Although the standard helmet with the solved dipoles found significantly less dipoles as compared to the standard helmet on the original dipoles (2.0 ± 0.4 and 2.2 ± 0.6, respectively, *p* < 0.005), the distance error was significantly larger (14.4 ± 11.0 and 12.4 ± 8.8 mm, respectively, *p* < 0.005).

Overall, selecting per each dipole pair a VMH that offers the best solution for its prior distribution, lead to improved distance accuracy in a feasible manner (yellow), and to move toward the results of the theoretically personalized VMH (purple) that can only be obtained by having full information regarding the actual characteristics of the dipoles. The latter suggests a ceiling effect of ∼57.5% improvement (that was obtained under the same constraints by the theoretically personalized VMH excluding the no-preference cases, or ∼46.6% otherwise: over all 100 cases) relative to standard helmet for these specific dipole pairs and examined set of VMHs (at the three-layer spherical grid resolution and the applied source localization method). The prior-based personalized VMH can come near this improvement.

## Discussion

This manuscript should be read as a proof of concept of the VMH method. Under the limitations of this study (listed in the relevant section below), the VMH method was assessed for source estimation accuracy relative to a standard MEG array. The results of this study showed that at low noise levels VMHs may significantly improve the distance accuracy of source estimations for the neuromagnetic fields that cannot be explained by a single generator. At theses low noise conditions, increasing the number of combined MEG arrays resulted in higher accuracy, particularly at large number of sources. Importantly, while low noise levels of 0.1 are unlikely in raw MEG data, they can be achieved by averaging similar signals.

Assuming the same resources of overall MEG recording time, the single standard MEG head position will include more events that can be averaged, compared to shorter recordings at several head positions. Accordingly, the VMH is characterized by a lesser noise suppression by a factor of the square root of the number of combined MEG arrays (i.e., head positions), that is if one supposes a similar event distributions and equal recording time in each head position (this factor was taken into account in the simulated noise, please see Section “Methods: Noise model”). At a high noise level, the source estimation accuracy was found to weaken by the VMHs, in comparison to a standard MEG helmet. The advantage in sampling did not overcome the shortage in noise suppression, and the specificity and sensitivity offered by VMHs deteriorated.

At low noise levels, that is for high SNR signals, the VMH may offer significant benefits. Enriching the spatial sampling of electromagnetic fields by constructing a VMH, and virtually increasing the effective gain of the measurement apparatus relative to the physical MEG helmet, may overcome the reduction in noise suppression and on average improve the source estimations. Nevertheless, even when no noise components are simulated (0 noise level), there were still cases where the standard helmet outperformed the VMHs. This demonstrates that the improvement obtained by the VMH concept is not a mere reflection of enhancing the SNR, and that the error of VMH estimation is not only noise driven.

Source estimation is a non-linear problem with an intrinsic sensitivity to the obtained spatial sampling of the generated electromagnetic fields. In some of the cases, the sampling by the add-on combined MEG arrays of the VMHs may in fact impair the reached solution. Therefore, it is important to optimize the VMH construct to the postulated sources, and thus to enrich the sampling in a custom-made way. While one can build a physical helmet with a sensor layout engineered to increase the rank of the gain matrix (i.e., optimize at general, for all grid points), this layout may not be optimal for specific neural generators (i.e., the same sensor layout can be the best for one neural pattern, and not the best for another). However, the advantage of the VMH concept is that it can be approximately optimized to the specific neural generators.

Based on evidence from previous MEG recording, that is assuming postulated sources, personalizing the construction of a VMH according to individual information was shown to be an advantageous approach. It seems that directing the changes in the head position with respect to the MEG sensor array, while relying on foregoing evidence, can improve source localization. Nonetheless, the personalization approach, as was introduced in this manuscript, is rather naïve, involving only a predefined set of VMHs, as well as assuming an identical recording time at each head position (i.e., emulated by the corresponding translated and rotated MEG array). This approach has proven useful, offering a ∼24.8% (or even a ∼36.4%, for cases that a VMH was in fact selected) improvement in source estimation accuracy in comparison to standard MEG for the cases and conditions studied, which suggest a potentially non-negligible contribution, if a relatively low noise level can be achieved (e.g., by averaging similar signals) and for complex sources that include at least two generators. To compare this improvement to figures known from EEG, for example, Song et al. (2015) demonstrated a more accurate source localization of EEG data by increasing the number of electrodes: a ∼27% improvement in localization distance error from 32 to 64 electrodes using minimum norm and full coverage, and only a ∼8 and ∼7% from 64 to 128 to 256, respectively. Moreover, the improvement demonstrated here was achieved by a two steps procedure: choosing a proper VMH based on the results from a previous recording in the standard head-MEG position and then conducting the new recordings and analyses according to the chosen VMH. However, the standard head position is only offered as a seemingly neutral position to begin with. An optimal VMH construct may not need to rely on this standard position. This should be examined in future studies. Moreover, given abundant recording time, a large number of steps, each in a different head position and duration, can be conducted, with a sequential improvement to the source estimations and hence to the chosen or constructed VMH. Hypothetically, this kind of procedure, which iteratively approximates the inverse problem solution, could serve to achieve a close to optimal solution.

### Application in epilepsy

Since the main clinical interest of the authors of this article is in the improvement of epilepsy surgery outcomes, the application of VMH in epilepsy is receiving special attention in the study. Nevertheless, the VMH approach is not limited to epilepsy and can be applied in other fields of basic and clinical neuroscience, such as estimation of brain activity hubs triggered by cognitive tasks. In epilepsy clinical practice, IEDs are commonly used to localize the irritative zone in patients suffering from epilepsy (Barkley, 2004; Bast et al., 2004; Pellegrino et al., 2016). When IEDs are averaged, high SNR is readily attainable, making the VMH method applicable and favorable. The magnitude of a typical interictal spike can be approximately a factor of 2 over background—at the peak of the spike the noise level will be close to 0.5 (a 1:2 ratio) and at half height it will be close to 1 (a 1:1 ratio); assuming an average of 100 spikes—the noise suppression will be by a factor of 10 (a square root of 100); therefore, at the peak - the noise level will be ∼0.05, and at half height - it will be ∼0.1. Moreover, the VMH benefits were demonstrated for complex fields associated with multiple generators. Markedly, by current methods, source estimation for complex fields, is challenging (Baillet et al., 2001; Hansen et al., 2010; Supek and Aine, 2019). The VMH approach can aid in such cases.

**FIGURE 7:**
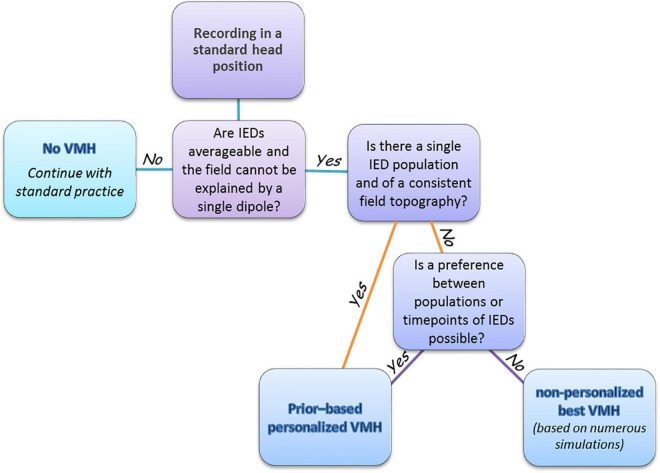
A decision scheme for implementing VMH approach in pre-surgery epilepsy clinic. After recording in a standard head position for 1 h, the clinician reviews the spread of the collected IEDs, and determines whether to: (1) continue with standard practice or (2) personalize the VMH, or (3) construct a VMH that generally works well for the specific MEG apparatus. The generally well VMH is pre-designed based on numerous simulations of randomly distributed dipoles.

Figure 7 depicts a suggested decision tree for the implementation of the VMH approach in the current clinical practice, as part of the presurgical evaluation of drug-resistant epilepsy patients. When seeking to determine the location of epileptogenic zone(s), first, it should be decided, based on data from preliminary standard MEG recording, whether the VMH approach can improve source estimation in the particular situation. If IEDs are not averageable (too few or too scattered) or if the field of averaged IED can be explained by a single dipole, the VMH is not a preferable approach and the recording should be continued according to regular clinical practice (Gross et al., 2013). Otherwise, benefit can be achieved by using either prior-based personalized or non-personalized VMH, as determined by the following set of conditions. If a single population of IEDs is present and their field topography is stable throughout the IED time course, then the personalized VMH approach is preferable. Notably, a single population of IEDs can be comprised of several neural generators at different locations, as is intended in this scenario. Likewise, the personalized VMH should be preferred if a decision can be made, whether: (1) one IED type is more important than others or (2) one time-point during the averaged IED time course [usually a half-way between IED start and peak (Lantz et al., 2003)] is more important than other time points. Both decisions can be based on data on main brain regions of interest achieved from other studies, such as video-EEG, MRI, PET. If preference cannot be made, the recommended approach is to utilize the head positions that comprise the best non-personalized VMH, given the sensor layout of the specific physical MEG apparatus. The best non-personalized VMH should be determined by numerous simulations of randomly distributed dipoles (within the scope of this study, and the limited set of simulated generators and examined VMHs, VMH no. 3, Table 1). Of note, while it is important to classify IEDs before averaging (Bast et al., 2004), the VMH approach can introduce pitfalls in the classification, since IEDs of the same type, measured in different head positions, have different field distribution through MEG sensors. However, simultaneously recorded EEG, which is not affected by head position, and utilizing the EEG-classified IEDs as triggers for the averaging of MEG signals may overcome this obstacle. Our suggested clinical decision algorithm can be feasibly implemented during a regular 2–3-h MEG session, when the first hour will be used to examine the characteristics of IEDs in standard MEG helmet recording, as a basis for deciding whether to continue the study with the regular helmet or with one of the VMHs. However, unrestrained recording time resources may permit the use of more sophisticated designs, including VMHs based on more than two MEG arrays, several differently personalized VMHs for distinct populations of IEDs, and long-duration recordings that can enable accumulating sufficient IEDs to optimize averaging.

### Limitations and future directions

The VMH concept offers a virtual rise in the number of sensors, and the distances and angles in which the sensors are placed relative to the electromagnetic field generators, enriching the spatial field sampling. Source estimation depends on the number and spatial distribution of sensors, yet, in conjunction with the selected forward model, the method of inverting the leadfield matrix, and the source space geometry (Baillet, 2010). Here, the simulations were carried out in simple settings (e.g., a spherical head model consisting of a three-layer grid, gaussian technical and physiological noise models, and SDF source estimation model). These choices were driven by owing clear and direct comparisons. For instance, SDF, is a non-linear model and is based on the sequential solution of over determined equation systems. SDF was chosen over competing methods, as it allowed to reduce the number of parameters used for comparison between the standard MEG array and VMHs to only two: the number of reconstructed sources and the distance between placed and estimated sources. This enabled a straightforward helmet preference. Moreover, SDF is considered here only as a tool for selecting the optimal field sampling strategy and not as a definitive source estimation method: after the optimal MEG array is selected, any inverse problem solution method can be used. The significant aspect of the applied source estimation technique is maintaining similar model attributes in all comparisons. In that regard, although committing what is sometime referred to as “the inverse crime” (Colton and Kress, 1998), using the same model for computing both forward and inverse solvers, the results for more than a single placed dipole are still not expected to be trivial (Wirgin, 2004). Furthermore, this choice supports comparisons between helmets by avoiding inserting artifacts that may be recovered from the particulars of the solvers’ representations (Wirgin, 2004). Additionally, in the carried-out simulations, sources were placed at random locations, with an equal probability throughout the grid. In practice, sources are not uniformly distributed. For example, in focal epilepsy there is a higher frequency for sources in the temporal lobe. Yet, MEG pre-surgical clinic often evaluates complex cases with extra-temporal sources. Random placements of sources were applied as a simplified approach to demonstrate the feasibility of the VMH concept. Overall, the conditions described above may limit the generalizability of the study conclusions. Future studies may aim to investigate the influence of other source estimation methods, particularly distributed approaches, and of more detailed forward models, including more realistic head models with a non-uniformly weighted (experimentally- or clinically- driven) source distribution, on the VMH concept.

The VMH approach is applicable to spatiotemporally stable sources and signals. Triggered or spontaneous recurring events, such as interictal epileptiform discharges (IED) or evoked responses, can benefit from the VMH. In contrast, VMH should not be applied to investigate ongoing activity or to analyze single events. The source estimation accuracy by a VMH may be influenced by various factors. Using VMH, it is important to confirm that the recorded brain activity, combined from different head positions, is related to the same generators. Accordingly, interictal epileptiform discharges should be correctly classified in different head positions using simultaneously recorded EEG. Failure to classify the signals correctly can lead to errors in source estimation. In the case of evoked responses, the habituation effect (Megela and Teyler, 1979) can lead to a decreased response amplitude in the second and/or later head positions. This phenomenon can also be detected by simultaneous EEG recordings. Temporal interruptions between MEG recoding in different head positions may eliminate the habituation effect. In addition, the VMH can fail to improve source estimation accuracy or even reduce it, if the data is too noisy or the amplitude of the signal is too low. In the case of an epilepsy patient with infrequent IEDs, the IED averaging can fail to reach a high SNR, which is needed for the VMH. The same can happen, when IEDs are too variable—in this case the signal classification will create many IED classes with low number of IEDs in every class. Finally, in the present work we demonstrated that VMH does not improve the source estimation accuracy if the magnetic field can be explained by only one equivalent current dipole.

The VMH method is unique for off-scalp MEG, but potentially can be expanded to other devices of flexible sensor layout. The personalized selection of a VMH based on a prior, can be further developed toward a fully tailored VMH procedure. Potential progresses in MEG machinery as well as in the applied algorithms can contribute to tailoring the VMH to the events and brain of interest, based on improving both (1) the prior of postulated source distribution and (2) the assembly of the VMH: refining the resolution in the head-MEG relative positions in parallel with adjusting the corresponding recording periods. Improving, at first stage, the established prior probability distribution of the neural generators, utilizing more advanced source localization techniques, which may also support distributed solutions, could further improve the VMH approach. In addition, future algorithms may possibly be iterative and online adaptive, to sequentially refine the source localization.

## Data availability statement

The original contributions presented in this study are included in the article/Supplementary material, further inquiries can be directed to the corresponding authors.

## Author contributions

OA and YH performed the simulations and analysis. OA wrote the first draft of the manuscript. DE and MM wrote sections of the manuscript. All authors contributed to conception and design of the study and manuscript revision, read, and approved the submitted version.

## Abbreviations

BN: brain noise
ECD: equivalent current dipole
IED: interictal epileptiform discharge
MEG: magnetoencephalography
ON: overall noise
SDF: sequential dipole fit
SNR: signal to noise ratio
SVD: singular value decomposition
TN: technical noise
VMH: virtual MEG helmet.

## References

[B1] Baillet S. (2010). “The dowser in the fields: Searching for MEG sources,” in *MEG an introduction to methods*, eds Hansen P. C. Kringelbach M. L. Salmelin R. (New York, NY: Oxford University press).

[B2] Baillet S. Mosher J. C. Leahy R. M. (2001). Electromagnetic brain mapping. *IEEE Signal Process. Mag.* 18 14–30. 10.1109/79.962275

[B3] Barkley G. L. (2004). Controversies in neurophysiology. MEG is superior to EEG in localization of interictal epileptiform activity: Pro. *Clin. Neurophysiol.* 115 1001–1009. 10.1016/j.clinph.2003.12.011 15066523

[B4] Bast T. Oezkan O. Rona S. Stippich C. Seitz A. Rupp A. (2004). EEG and MEG source analysis of single and averaged interictal spikes reveals intrinsic epileptogenicity in focal cortical dysplasia. *Epilepsia* 45 621–631. 10.1111/j.0013-9580.2004.56503.x 15144427

[B5] Berger H. (1929). Über das Elektrenkephalogramm des Menschen. *Arch. Psychiatr. Nervenärzte.* 87 527–570. 10.1007/BF01797193

[B6] Cohen D. (1968). Magnetoencephalography: Evidence of magnetic fields produced by alpha-rhythm currents. *Science* 161 784–786. 10.1126/science.161.3843.784 5663803

[B7] Colton D. Kress R. (1998). *Inverse acoustic and electromagnetic scattering theory*, Vol. 93. Berlin: Springer. 10.1007/978-3-662-03537-5

[B8] Cuffin B. N. Cohen D. (1977). Magnetic fields of a dipole in special volume conductor shapes. *IEEE Trans. Biomed. Eng.* 24 372–381. 10.1109/TBME.1977.326145 881208

[B9] Gross J. Baillet S. Barnes G. R. Henson R. N. Hillebrand A. Jensen O. (2013). Good practice for conducting and reporting MEG research. *Neuroimage* 65 349–363. 10.1016/j.neuroimage.2012.10.001 23046981 PMC3925794

[B10] Hämäläinen M. Hari R. Ilmoniemi R. J. Knuutila J. Lounasmaa O. V. (1993). Magnetoencephalography—theory, instrumentation, and applications to noninvasive studies of the working human brain. *Rev. Mod. Phys.* 65:413. 10.1103/RevModPhys.65.413

[B11] Hansen P. C. Kringelbach M. L. Salmelin R. (2010). *MEG: An introduction to methods.* New York, NY: Oxford Univ Press.

[B12] Iivanainen J. Mäkinen A. Zetter R. Stenroos M. Ilmoniemi R. J. Parkkonen L. (2019). Sampling theory for spatial field sensing: Application to electro- and magnetoencephalography. *arXiv* [Preprint]. arXiv:1912.05401

[B13] Iivanainen J. Mäkinen A. J. Zetter R. Stenroos M. Ilmoniemi R. J. Parkkonen L. (2021). Spatial sampling of MEG and EEG based on generalized spatial-frequency analysis and optimal design. *Neuroimage* 245:118747. 10.1016/j.neuroimage.2021.118747 34852277 PMC8752968

[B14] Kohn M. Senyak J. (2020). *Sample size calculators [website].* UCSF CTSI. Available online at: https://www.sample-size.net/ (accessed November 22, 2020).

[B15] Lantz G. Spinelli L. Seeck M. De Peralta Menendez R. G. Sottas C. C. Michel C. M. (2003). Propagation of interictal epileptiform activity can lead to erroneous source localizations: A 128-channel EEG mapping study. *J. Clin. Neurophysiol.* 20 311–319. 10.1097/00004691-200309000-00003 14701992

[B16] Lindfield G. Penny J. (2019). “Linear equations and eigensystems,” in *Numerical methods*, eds Lindfield G. Penny J. (Amsterdam: Elsevier), 73–156. 10.1016/b978-0-12-812256-3.00011-7

[B17] Medvedovsky M. Nenonen J. Koptelova A. Butorina A. Paetau R. Makela J. P. (2016). Virtual MEG helmet: Computer simulation of an approach to neuromagnetic field sampling. *IEEE J. Biomed. Health Inform.* 20 539–548. 10.1109/JBHI.2015.2392785 25616085

[B18] Medvedovsky M. Taulu S. Gaily E. Metsähonkala E.-L. L. Mäkelä J. P. Ekstein D. (2012). Sensitivity and specificity of seizure-onset zone estimation by ictal magnetoencephalography. *Epilepsia* 53 1649–1657. 10.1111/j.1528-1167.2012.03574.x 22780219

[B19] Megela A. L. Teyler T. J. (1979). Habituation and the human evoked potential. *J. Comp. Physiol. Psychol.* 93 1154–1170. 10.1037/h0077630 521525

[B20] Nurminen J. Taulu S. Nenonen J. Helle L. Simola J. Ahonen A. (2013). Improving MEG performance with additional tangential sensors. *IEEE Trans. Biomed. Eng.* 60 2559–2566. 10.1109/TBME.2013.2260541 23649129

[B21] Nurminen J. Taulu S. Okada Y. (2010). Improving the performance of the signal space separation method by comprehensive spatial sampling. *Phys. Med. Biol.* 55 1491–1503. 10.1088/0031-9155/55/5/015 20157231

[B22] Oostenveld R. Fries P. Maris E. Schoffelen J.-M. (2011). FieldTrip: Open source software for advanced analysis of MEG, EEG, and invasive electrophysiological data. *Comput. Intell. Neurosci.* 2011:156869. 10.1155/2011/156869 21253357 PMC3021840

[B23] Pellegrino G. Hedrich T. Chowdhury R. Hall J. A. Lina J.-M. Dubeau F. (2016). Source localization of the seizure onset zone from ictal EEG/MEG data. *Hum. Brain Mapp.* 37 2528–2546. 10.1002/hbm.23191 27059157 PMC6867380

[B24] Salmelin R. (2010). “Multi-dipole modelling in MEG,” in *MEG: An introduction to methods*, eds Hansen P. C. Kringelbach M. L. Salmelin R. (Oxford: Oxford University Press).

[B25] Sarvas J. (1987). Basic mathematical and electromagnetic concepts of the biomagnetic inverse problem. *Phys. Med. Biol.* 32 11–22. 10.1088/0031-9155/32/1/004 3823129

[B26] Shannon C. E. (1949). Communication in the presence of noise. *Proc. IRE* 37 10–21. 10.1109/JRPROC.1949.232969

[B27] Song J. Davey C. Poulsen C. Luu P. Turovets S. Anderson E. (2015). EEG source localization: Sensor density and head surface coverage. *J. Neurosci. Methods* 256 9–21. 10.1016/j.jneumeth.2015.08.015 26300183

[B28] Srinivasan R. Tucker D. M. Murias M. (1998). Estimating the spatial Nyquist of the human EEG. *Behav. Res. Methods Instrum. Comput.* 30 8–19. 10.3758/BF03209412

[B29] Supek S. Aine C. J. (2019). *Magnetoencephalography: From signals to dynamic cortical networks*, 2nd Edn. Cham: Springer. 10.1007/978-3-030-00087-5

[B30] Tierney T. M. Mellor S. O’Neill G. C. Holmes N. Boto E. Roberts G. (2020). Pragmatic spatial sampling for wearable MEG arrays. *Sci. Rep.* 10:21609. 10.1038/s41598-020-77589-8 33303793 PMC7729945

[B31] Vrba J. Robinson S. E. McCubbin J. (2004). How many channels are needed for MEG? *Neurol. Clin. Neurophysiol.* 2004:99. 16012656

[B32] Wirgin A. (2004). The inverse crime. *arXiv* [Preprint]. arXiv:math-ph/0401050

