# Supplementary material for: Source localization using virtual magnetoencephalography helmets: A simulation study toward a prior-based tailored scheme
Source: Front Neurosci. 2022 Sep 6;16:947228. doi: 10.3389/fnins.2022.947228 (PMC9485615; doi:10.3389/fnins.2022.947228)
Supplement: Supplementary file 1 [file Data_Sheet_1.PDF]

Appendix

Inverse model: determining the threshold

In the current study, sequential dipole fit (SDF) (*i.e.*, sequential single ECD fit) has been used in source estimation. Successively, in six iterative steps, each single ECD was fitted to the (residual) field. Dipoles with a magnitude (Euclidean norm) that is proportionally weaker than a threshold of 0.3 of strongest dipole were discarded. The choice of 0.3 threshold, was based on examining several potential values. Fig. S1 - top row, displays an example for 3 placed dipoles at 0.1 noise level: Undoubtedly, the number of solved dipoles decreases as the threshold increases (a significant main effect for threshold: p as small as precision limit), while there was no effect for the number of combined MEG arrays ($p>0.3$), and the significant interaction effect between threshold and number of arrays ($p<0.02$) was not significant for the same threshold at different number of combined MEG arrays ($p>0.1$). Similar trends were demonstrated for different number of placed dipoles and at low noise levels in general, while at high noise levels, beside the same clear effect of the selected value of the threshold (the number of solved dipoles increases as the threshold decreases, p as small as precision limit), there was also a significant increase in number of solved dipoles as the number of combined MEG arrays increased ($p<10^{-25}$), and a significant interaction effect ($p<10^{-8}$) but only for either 0.1 or 0.2 thresholds when comparing the same threshold and as the number of combined MEG arrays increased ($p<0.03$). Moreover, at both low and high noise levels there were no significant interaction effects between threshold and number of combined MEG arrays ($p>0.8$) (data not shown).

Quantitative assessment of source localization: maximal distance error

In the current study, the mean distance accuracy between the locations of placed and solved dipoles was calculated, as a means to compare the source estimations obtained by a standard MEG helmet and VMHs. Notably, the significant differences in distance accuracy should not be assigned to a difference in the maximal error, which if so would have suggested a failure in the association between placed and solved dipoles. For instance: Fig. S2, bottom row, right panel demonstrates an example, for 3 placed dipoles and at 0.1 noise level, that the maximal distance error between placed and solved dipoles does not significantly varies: no significant effect for the number of combined MEG arrays ($p>0.6$) and no significant interaction effect between number of arrays and number of placed dipoles ($p>0.8$)).

Simulations part I: statistics

When comparing source estimations by standard helmet to VMH (Fig. 2), the effects of both the number of arrays and the number of placed dipoles on the distance error or the number of solved dipoles were examined by a two-way ANOVA tests.

- At low noise levels (0 or 0.1):

  The distance error -

  - A significant interaction effect "number of arrays"*"number of placed dipoles" ($p<10^{-6}$ and $p<10^{-8}$, for no noise and 0.1 noise levels, respectively), particularly for the same number of placed dipoles, there were significant effects for 2 placed dipoles, between 1 and 3 arrays p=0.0030 and 0.0083 for no noise and 0.1 noise level, respectively; for 3 placed dipoles, between 1 and 3 arrays $p<10^{-6}$ for both no noise and 0.1 noise levels; and for 4 and 5 placed dipoles, between all number of arrays p was in the range of between 0.01 till 10^{-6}, excluding a non-significant effect between 2 and 3 arrays (for 5 placed dipoles p=0.18 and 0.12, and for 4 placed dipoles at 0.1 noise level p=0.39).
  - A significant main effect for number of arrays ($p<10^{-30}$ and $p<10^{-22}$, respectively).
  - A significant main effect for number of placed dipoles (p as small as precision limit for all noise levels).

  The number of solved dipoles –

  - A non-significant interaction effect "number of arrays"*"number of placed dipoles" at low noise levels (p=0.44, 0.20 for no noise and 0.1 noise levels, respectively) was demonstrated, particularly for the same number of placed dipoles, there was no significant effect for different number of arrays (p in the range of 0.16 till 1).
  - A significant main effect for number of arrays (p=0.037, and 0.035, for no noise and 0.1, respectively).
  - A significant main effect for number of placed dipoles (p as small as precision limit for all noise levels).

- At high noise levels (0.3):

  The distance error –

  - A significant interaction effect ($p<10^{-12}$), particularly for the same number of placed dipoles, there were significant effect for 1 placed dipole, between 1 and 3 and 1 and

2 arrays $p<10^{-5}$ and for 2 placed dipoles, between 1 and 3 arrays p=0.0012.

- A significant main effect for number of arrays ($p<10^{-8}$)
- A significant main effect for number of placed dipoles (p as small as precision limit)

The number of solved dipoles –

- A significant interaction effect ($p<10^{-14}$): for 1 or 2 placed dipoles, there was a significant effect for different number of arrays (p<0.05 higher as the number of arrays increased, excluding for 2 placed dipoles between 1 and 2 arrays p=0.35), while for 3 to 5 placed dipoles, there was no significant effect for different number of arrays (p in the range of 0.14 till 1).
- A significant main effect for number of arrays ($p<10^{-34}$)
- A significant main effect for number of placed dipoles (p as small as precision limit)

- The effect of noise levels:

As an example Fig. S1 (middle row) depicts the case of 3 placed dipoles. Similar trends were observed for other numbers of placed dipoles. The significant interaction effect between noise level and number of combined MEG arrays for both in distance accuracy ($p<10^{-33}$) and number of solved dipoles ($p<10^{-55}$) is such that the trend at low noise levels (between 0 till 0.1) is different than the trend for high noise levels (0.3 and above)– at low noise level the distance error significantly decrease between standard helmet and 3 combined arrays (p<0.001), and no significant difference in the number of solved dipoles (p>0.5), while at high noise levels: for 0.3 noise level the difference in distance error for dissimilar number of combined arrays is not significant (p>0.3), and for higher noise levels significant for standard helmet versus the VMHs ($p<10^{-5}$), and also a significant difference for the number of solved dipoles for all noise levels above 0.3 and for dissimilar number of combined MEG arrays (p<0.01, excluding for 0.3 noise level between 1 and 2 combined arrays). (Comment: as the threshold for declaring an active source was set to 0.3, at high noise levels there are noise components which lead to finding superfluous dipoles).

Figure S1.

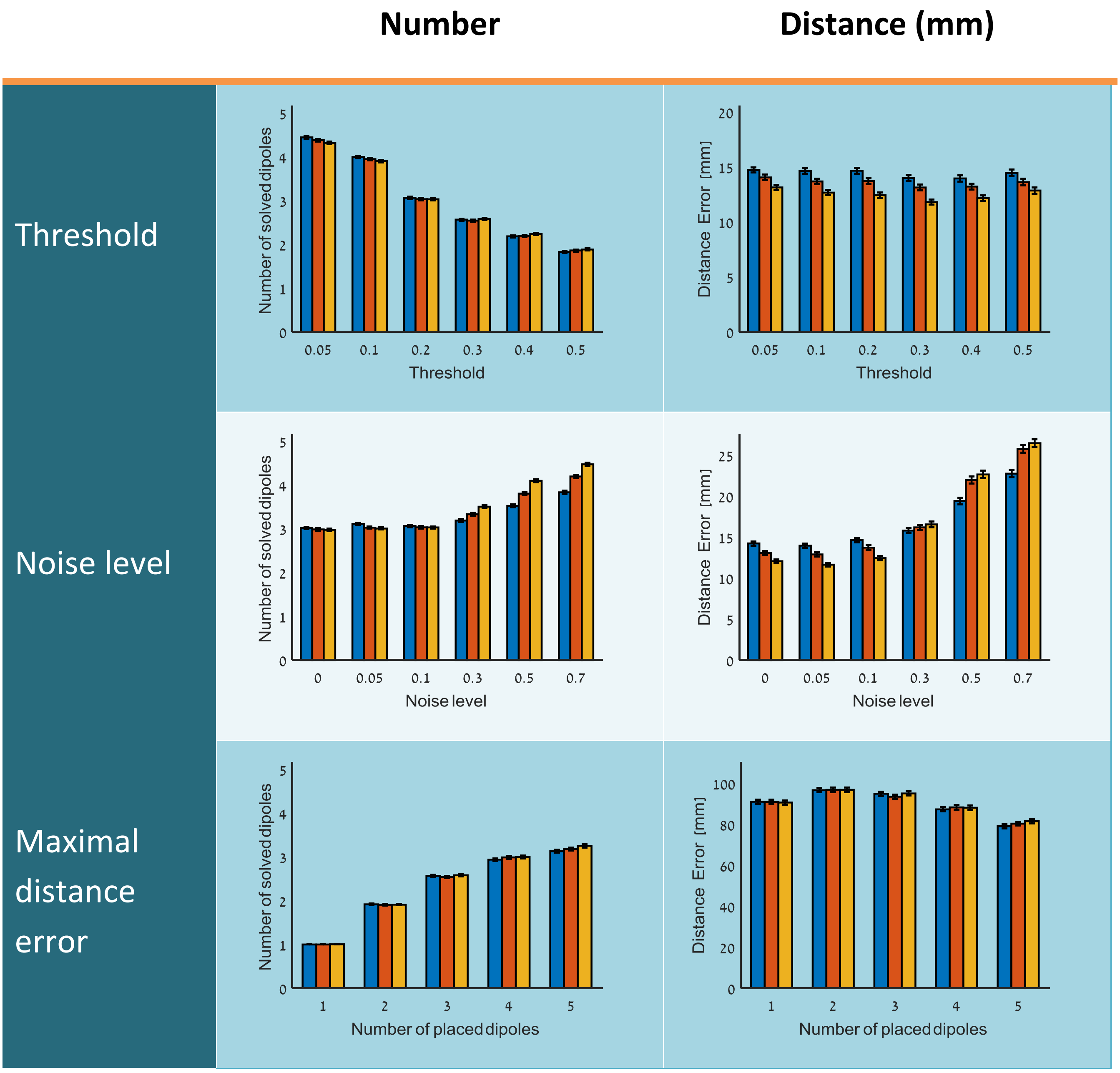

Fig. S1 ***Exploring the contribution of various factors on source estimation accuracy***. The number (left column) and the distance accuracy (right column) for number of placed dipoles equal 3, as an example. The influence of the following factors is presented: (A) Threshold (at noise level equal 0.1) (B) Noise level. (C) Maximal error in distance between solved and placed dipoles (at noise level equal 0.1). Left panel in C is identical to Fig. 2, top row, middle column. Each bar represents a mean ± SEM across 1000 simulations.

Figure S2.

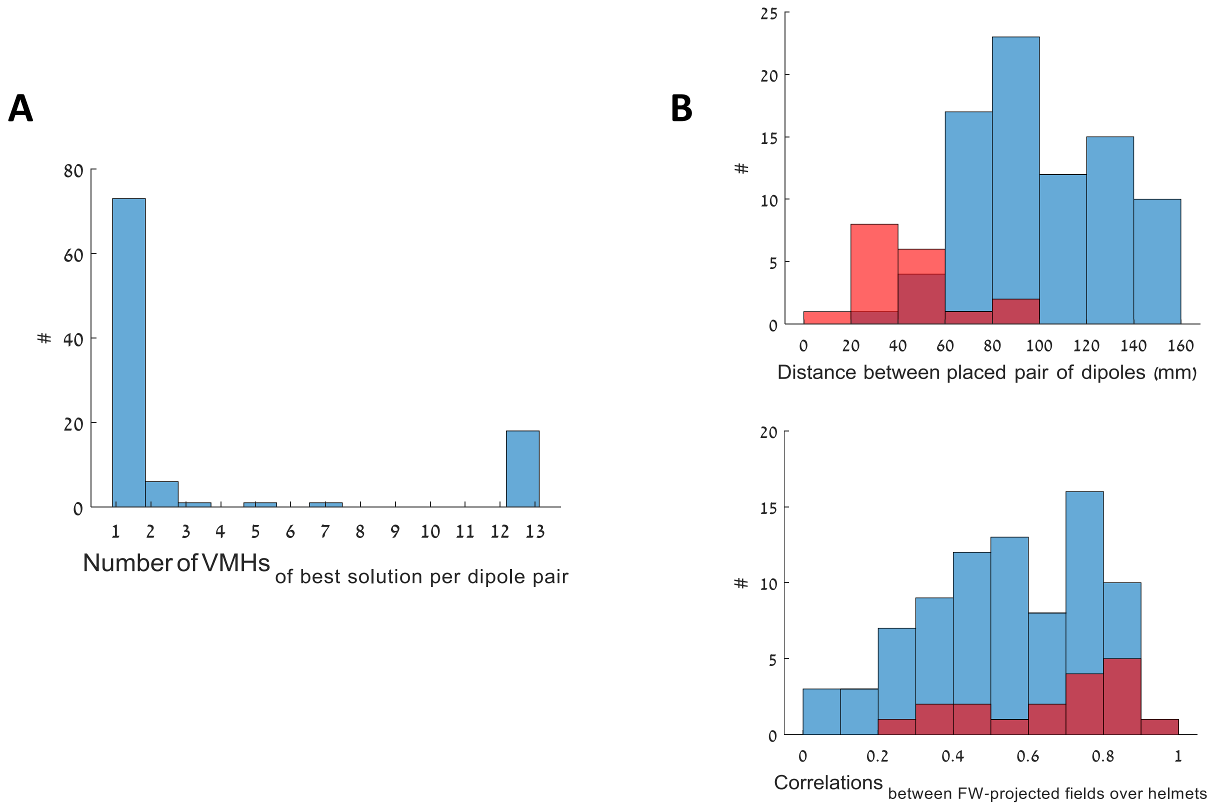

Fig. S2 ***Personalizing a VMH to a dipole pair: excluded cases***. Based on a prior of source estimations by simulated previous recording, a VMH is selected, personalized to those estimated sources. (A) A distribution of the number of VMHs that were able to offer the best solution out of the evaluated VMHs (Fig. 3A) per each of the 100 dipole pairs examined. As the distribution demonstrates, in 18 cases all VMHs offered a solution with the same source estimation quality, hence, no preferable VMH was found. In 73 of the cases, there was 1 specific VMH that outperform the other VMHs per the specific dipole pair. In the other 9 cases, the prior-based VMH was chosen at random from the candidate VMHs which preform equally well. (B) Distributions of all cases that there was a VMH or several (less than all) that offered the best solution (blue) and of the 18 cases, where there was not a preferable VMH (red): the distance between the dipole pairs in these cases (left panel) and the correlations between the forward projected fields over the MEG arrays comprising the VMHs (right panel) are significantly different between the distribution (using a permutation test: $p=0.0001$ and $p<0.05$, respectively).
